# The Endosteal Niche in Breast Cancer Bone Metastasis

**DOI:** 10.3389/fonc.2020.00335

**Published:** 2020-03-13

**Authors:** Marie-Therese Haider, Daniel J. Smit, Hanna Taipaleenmäki

**Affiliations:** ^1^Molecular Skeletal Biology Laboratory, Department of Trauma, Hand and Reconstructive Surgery, University Medical Center Hamburg-Eppendorf, Hamburg, Germany; ^2^Institute of Biochemistry and Signal Transduction, University Medical Center Hamburg-Eppendorf, Hamburg, Germany

**Keywords:** breast cancer, bone metastases, endosteal niche, microenvironment, osteoblast, fibroblast

## Abstract

The establishment of bone metastasis remains one of the most frequent complications of patients suffering from advanced breast cancer. Patients with bone metastases experience high morbidity and mortality caused by excessive, tumor-induced and osteoclast-mediated bone resorption. Anti-resorptive treatments, such as bisphosphonates, are available to ease skeletal related events including pain, increased fracture risk, and hypercalcemia. However, the disease remains incurable and 5-year survival rates for these patients are below 25%. Within the bone, disseminated breast cancer cells localize in “metastatic niches,” special microenvironments that are thought to regulate cancer cell colonization and dormancy as well as tumor progression and subsequent development into overt metastases. Precise location and composition of this “metastatic niche” remain poorly defined. However, it is thought to include an “endosteal niche” that is composed of key bone cells that are derived from both, hematopoietic stem cells (osteoclasts), and mesenchymal stromal cells (osteoblasts, fibroblasts, adipocytes). Our knowledge of how osteoclasts drive the late stage of the disease is well-established. In contrast, much less is known about the interaction between osteogenic cells and disseminated tumor cells prior to the initiation of the osteolytic phase. Recent studies suggest that mesenchymal-derived cells, including osteoblasts and fibroblasts, play a key role during the early stages of breast cancer bone metastasis such as tumor cell homing, bone marrow colonization, and tumor cell dormancy. Hence, elucidating the interactions between breast cancer cells and mesenchymal-derived cells that drive metastasis progression could provide novel therapeutic approaches and targets to treat breast cancer bone metastasis. In this review we discuss evidences reporting the interaction between tumor cells and endosteal niche cells during the early stages of breast cancer bone metastasis, with a particular focus on mesenchymal-derived osteoblasts and fibroblasts.

## Introduction

Metastasis is a complex, multi-step process during which cancer cells escape from the primary tumor, circulate, disseminate to the distant organs, and eventually colonize and grow in the metastatic site (1). One of the essential steps in metastases development is the ability of cancer cells to adapt to the new environment which is very different from the environment in the tissue of origin. The interaction between cancer cells and the metastatic environment was already proposed in 1889 by Sir Stephen Paget who suggested that metastatic colonization of a distant organ is not a random process and that cancer cells can only grow in a supportive microenvironment (2). This so called “Seed and Soil” theory in which the cancer cells are the seeds and the bone is the soil can be considered as the first evidence of the “niche” concept. Nevertheless, more than a century later we are still in process of understanding the complex interaction between the cancer cells and the local and metastatic microenvironments or “niches.”

Bone metastases involve complex interactions between the cancer cells and the cells of the bone microenvironment, including endothelial cells, hematopoietic stem cells (HSCs), mesenchymal stromal cells (MSCs), and bone cells (bone forming osteoblasts and bone resorbing osteoclasts) (3). The role of osteoclasts in driving the progression of breast cancer bone metastases is well-established (4). During the so called “vicious cycle of bone metastases” osteoclasts are activated directly or indirectly by the tumor cells (5). Increased osteoclast function results in pathological bone resorption during which several growth factors, including transforming growth factor β (TGF-β) are released from the bone matrix. These factors support tumor growth and further osteoclast activation (6). In contrast, the contribution of osteoblasts to disease establishment has been underappreciated and poorly investigated. However, recently research has moved away from the concept that osteoclasts alone drive the progression of breast cancer bone metastasis and osteoblasts are more and more investigated as novel cellular targets (7, 8). In order to develop novel, more successful therapies to prevent or treat cancer-induced bone disease, a better understanding of the interaction of tumor cells and cells of the bone microenvironment is required, in particular the tumor—bone cell communications prior to the formation of osteolytic lesions. In the following chapters we will discuss the role of bone marrow niches, in particular the endosteal niche, in the development and progression of bone metastasis as well as the function of osteoblasts and fibroblasts in this process.

## Bone Marrow Niches

### Physiological Niche

In bone, the physiological niche is composed of several local environments including the endosteal niche and the vascular niche (9). The endosteal niche lines the trabecular and endocortical bone surface and consists of osteoblasts that form new bone and osteoclasts that resorb the bone. Osteoblasts are derived from MSCs in a process tightly controlled by various transcription factors and signaling pathways. The key transcription factors Runx2 and osterix (Osx) promote MSC commitment to osteoprogenitors and further differentiation to mature osteoblasts (10). Mature osteoblasts secrete bone matrix proteins including collagen I (Col1), alkaline phosphatase (ALP) and osteocalcin, and contribute to bone formation. Mature osteoblasts can be embedded in the bone matrix as osteocytes that function as mechanosensory cells and contribute to bone remodeling (11). Alternatively, osteoblasts can adapt a quiescent state on the bone surface as bone lining cells or undergo apoptosis. Osteoblast differentiation is promoted by various paracrine factors including parathyroid hormone (PTH) and wingless (Wnt) proteins that activate the respective signaling pathways (11, 12). Besides osteoblasts, MSCs can give rise to other mesenchymal cell populations including adipocytes, chondrocytes and myocytes. Adipocytes are a frequent cell type in the bone marrow and an inverse relationship has been shown to occur between osteogenesis and adipogenesis of MSCs (13).

Bone-resorbing osteoclasts are multinucleated cells of hematopoietic origin. Osteoclast differentiation is supported by various cytokines including the macrophage colony-stimulating factor (MCS-F) and the receptor activator of nuclear factor kappa-B ligand (RANKL) that are produced by osteoblasts (14). In turn, osteoclasts secrete factors such as Wnt 10b, sphingosine-1-phosphate and bone morphogenic protein 6 (BMP-6) to regulate osteoblast differentiation and function. Additionally, bone matrix-derived factors including but not limited to TGF-ß, insulin like growth factors (IGFs) and bone morphogenic proteins (BMPs) are released during osteoclast-mediated bone resorption and can modify osteoblast progenitors. Detailed coupling mechanisms between osteoblasts and osteoclasts are reviewed in (15). Through these coordinated actions bone formation and resorption are often coupled under physiological conditions. In addition to its role in bone remodeling, the endosteal niche has been proposed to maintain hematopoietic stem cells (HSCs) in a quiescent state.

The vascular niche consists of endothelial cells, closely located pericytes and smooth muscle cells. The vascular niche is important for stem and progenitor cell function. Through secretion of angiocrine growth factors, the vascular niche recruits endothelial progenitors, MSCs and HSCs (16, 17). In contrast to the endosteal niche that supports HSC quiescence, the vascular niche has been shown to promote HSC mobilization, proliferation and differentiation and thus the activation of HSCs (17). Although the endosteal and vascular niches can be considered as independent microenvironments their interaction is crucial for various physiological functions including HSC maintenance and coupling of angiogenesis and osteogenesis (17, 18).

### Pre-metastatic Niche

In cancer, the physiological functions of the niches are hijacked by metastatic cancer cells. Cancer cells alter the niche to support their own functions from tumor cell dissemination to dormancy, relapse, and growth. Importantly, the first changes in the expression of the components of the extracellular matrix (ECM) and mobilization of bone marrow progenitor cells occur already before the cancer cells arrive in the metastatic site such as the bone marrow or the lung (19). Preparation of this so-called “pre-metastatic niche” creates a conductive microenvironment for the cancer cells that eventually disseminate to distant organs.

Formation of the pre-metastatic niche requires remodeling of the ECM and deposition of aberrant ECM. Important ECM proteins include fibronectin, tenascin and periostin that form fibrillar networks and regulate cancer cell adhesion and growth (19, 20). Among other factors, breast cancer cells in the primary tumor secrete lysyl oxidase (LOX) that regulates fibronectin activity and matrix remodeling (21). LOX also alters the endosteal niche by activating the osteoclasts, thus preparing a permissive environment for circulating tumor cells to colonize the bone (22). Interestingly, high LOX expression in the primary tumor is associated with bone metastases without affecting the primary tumor growth. Recently, tumor exosomes have been shown to prepare the pre-metastatic niche and direct organotrophic metastasis through the expression of diverse integrins (23).

### Metastatic Niche

**Figure 1:**
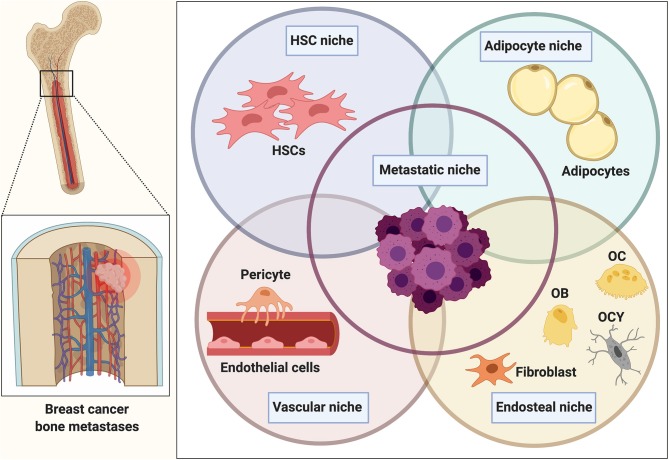
The bone metastatic niche. Once homed to bone, tumor cells are exposed to a heterogeneous microenvironment that is comprised of various individual cellular entities. The complex interplay between osteoblasts and osteoclasts during bone remodeling in addition to the presence of various other bone marrow-derived populations makes the bone microenvironment a favorable and supportive environment (metastatic niche) for disseminated cancer cells. Within bone, the metastatic niche is thought to be comprised of a hematopoietic stem cell niche (HSCs), endosteal (osteoclasts (OC), osteoblasts (OB), osteocytes (OCY), fibroblasts), and vascular niche (endothelial cells, pericytes). Several findings also implicate a role of the bone marrow adipocyte niche in bone metastasis. The interaction and overlap between the niches remain to be determined and resulted in the generalized term of the “metastatic niche” that is thought to regulate homing, survival and dormancy of tumor cells.

Within bone, the proposed metastatic niche is composed of several individual and distinct cellular entities comprising a hematopoietic, endosteal, and vascular niche (Figure 1). Emerging evidence also implicates a role for the bone marrow adipocyte niche in bone metastasis (24, 25). These niches are thought to determine the fate of disseminated tumor cells (DTCs), namely whether they will actively proliferate, stay quiescent/dormant or die. Breast cancer dissemination to the bone has been shown to occur E-selectin-mediated interactions in the sinusoidal regions (vascular niche) (26). The sinusoidal vasculature also regulates HSC transit through the same mechanism and once in the bone, cancer cells have been proposed to compete with HSCs for their niche.

Both the vascular and the endosteal niche have been shown to maintain breast cancer cell dormancy though different cues (27, 28). The vascular niche has been proposed to function as a “pro-dormancy” niche maintaining the cancer cells quiescent. The niche-derived molecules regulating cancer cell dormancy include a chemokine stromal cell-derived factor 1 [SDF-1, also known as C-X-C motif chemokine ligand 12 (CXCL12)] that binds to its receptor C-X-C chemokine receptor type 4 (CXCR4) on cancer cells and anchors the cancer cells in the niche (26). In addition, thrombospondin expressed by endothelial cells in a stable microvasculature has been shown to induce breast cancer cell quiescence (27). While a stable vasculature promotes dormancy, active sprouting neovasculature has been proposed to release cancer cells from the dormant state and support micrometastases growth via TGF-β and periostin (27).

In addition to the vascular niche and the endothelial niche, the adipocytes have been proposed as important players of the metastatic niche. Bone marrow adipocity increases during aging and thus, the potential role of the adipogenic niche becomes increasingly important in the elderly suffering from breast cancer. Indeed, in a model of human bone tissue, breast cancer cells were shown to migrate into the bone marrow adipose tissue and establish direct cellular interactions with the adipocytes (24). The recruitment was shown to be mediated by adipose-derived leptin and interleukin (IL)-1β, highlighting the role of cytokines and adipokines in breast cancer bone colonization.

## Osteoblasts in Tumor Cell Homing, Dissemination, and Dormancy

**Figure 2:**
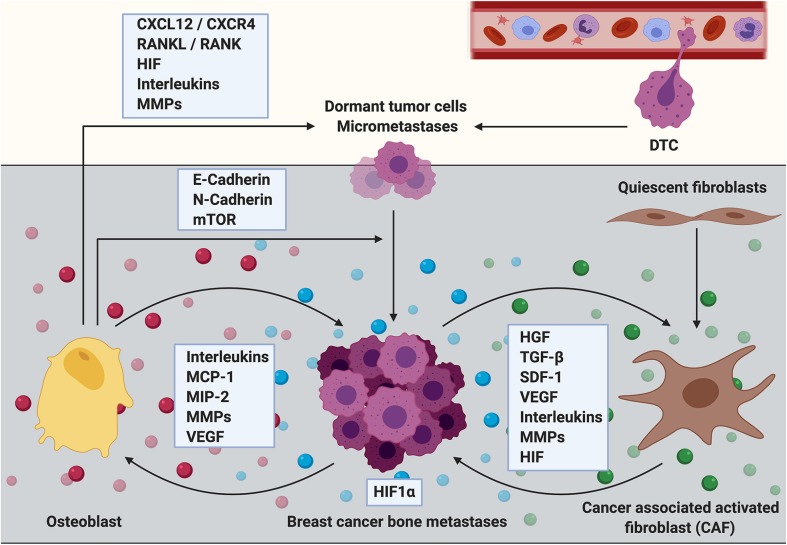
The role of osteoblasts and cancer associated fibroblasts during the establishment and progression of breast cancer bone metastasis. Cells of mesenchymal origin including osteoblasts and cancer associated fibroblasts (CAFs) are increasingly recognized to contribute to the establishment and progression of breast cancer bone metastasis. Osteoblasts express cytokines including C-X-C motif chemokine ligand 12 (CXCL12, also referred to as stromal derived factor 1, SDF-1) and receptor activator of NF-κB ligand (RANKL) that promote breast cancer cell dissemination and metastatic growth through interaction with their corresponding receptors (CXCR4 and RANK, respectively) that are expressed by the cancer cells. Breast cancer micrometastases have also been shown to be surrounded by osteoblastic cells. The interaction between breast cancer cells and osteoblasts could partially be mediated via heterotypic adherens junctions using E-, and N-cadherins resulting in an enhanced mTOR activity in cancer cells and consequently in the transition from dormant tumor cells into overt metastases. Osteoblasts also express high levels of extracellular matrix remodeling proteins (MMPs) in addition to reduced presence of inflammatory cytokines such as interleukins (ILs) upon cancer cell stimulation. Thereby osteoblasts could regulate breast cancer cell dormancy in the bone microenvironment. In contrast, metastatic breast cancer cells increase the production of inflammatory cytokines such as IL-6 and IL-8, monocyte chemoattractant protein−1 (MCP-1), macrophage—inflammatory protein 2 (MIP-2) and vascular endothelial growth factor (VEGF) in osteoblasts, thereby promoting breast cancer cell invasiveness and metastasis progression. Although usually quiescent in normal tissue, fibroblasts acquire an activated phenotype during processes such as wound healing or inflammation. Activated fibroblasts in the tumor stroma are called cancer associated fibroblasts (CAFs). They produce growth factors that contribute to disease progression including hepatocyte growth factor (HGF), transforming growth factor beta (TGF-β), stromal derived factor 1 (SDF-1 or CXCL12), VEGF, IL-6, and other ILs in addition to MMPs. All of these factors promote primary tumor growth and it can be hypothesized that CAFs could similarly mediate the growth of breast cancer bone metastases. CAFs are known to induce extracellular matrix remodeling and alter the stiffness of tissues thereby facilitating tumor cell invasion, dissemination and/or metastasis. CAF-induced matrix remodeling and CAF invasion have been shown to be supported by hypoxia inducible factor−1 alpha (HIF1α). In turn, an increased expression of HIF1α might stimulate the tumor growth promoting function of CAFs.

Osteoblasts are also suggested as potential mediators of breast cancer cell homing to bone (Figure 2). This arises from the observations that disseminated breast cancer cells are frequently found in bone areas that are rich in osteoblasts (29). This phenomenon could in part be mediated by the fact that osteoblasts express SDF-1 and RANKL, two cytokines that favor breast cancer cell dissemination and ultimately tumor growth through binding to their cognate receptors (CXCR4 and receptor activator of nuclear factor kappa-B (RANK), respectively) on the cancer cells (26, 30). Hypoxia-inducible factor (HIF)-signaling in osteoprogenitor cells for example has been shown to not only promote metastasis in the bones, but to also stimulate breast cancer cell dissemination to organs beyond the skeleton, such as for example the lung, partially through the production of SDF-1 (31). The hypothesis that tumor cells use the SDF-1/CXCR4 axis to hone to the osteoblastic niche in bone is supported by the finding that both, newly and established metastases are anchored in the bone marrow by SDF-1/CXCR4 interactions (26). Already in 2006, Phadke and colleagues reported that the majority of disseminated MDA-MB-435 breast cancer cells located in the primary spongiosa of the metaphysis of the distal femur, where metastatic growth ultimately proceeded (32). Furthermore, in an intracardiac model using BALB/c nude mice MDA-MB-231 breast cancer cells preferentially localized in the metaphysis, and especially close to trabecular bone surfaces that are rich in osteoblasts (29). Consistently, early metastases of a breast cancer cell line obtained from MMTV-PyMT mice were found adjacent to trabecular bone areas below the growth plate cartilage that was enriched in osteoprogenitor cells (OPN^high^, SDF-1^high^) (31). The metaphysis might provide a rich reservoir of growth factors, especially through the dense, interconnected vascular system (33). Although there might be a differential expression of adhesion molecules and growth factors in this area, studies have also shown that Runx2, Col1α, and Osx-positive osteoblasts are abundantly located around CD31-positive bone marrow vessels in the metaphysis (33).

Once in the bone, tumor cells can remain dormant for decades until the development of metastatic disease. Importantly, it still remains unknown what triggers the initiation from dormant DTCs or micrometastases into actively proliferating metastases. It has been suggested that osteoblasts, upon the presence of breast cancer cells, might produce soluble factors that act as chemoattractants, maintenance and/or growth factors for both, breast cancer cells and/or osteoclasts. Consequently, this would result in the activation of the vicious cycle of bone metastasis and osteolytic disease (34). Studies by Kinder and colleagues report that metastatic MDA-MB-231 breast cancer cells increase the production of inflammatory cytokines such as IL-6, monocyte chemoattractant protein-1 (MCP-1), and IL-8 in both, human hFOB 1.19, and murine M3T3-E1 osteoblasts (35). Similar results are documented by Bussard and colleagues showing an increased presence of osteoblast-derived cytokines including IL-6, IL-8, MCP-1, macrophage-inflammatory protein 2 (MIP-2), and vascular endothelial growth factor (VEGF) in the presence of MDA-MB-231 breast cancer cells in *ex vivo* cultures of tumor bearing bones from athymic mice, or even in the presence of conditioned medium *in vitro* (34). MCP-1 for example is known to be involved in osteoclastogenesis as well as in the attraction and infiltration of monocytes and macrophages during inflammation (36). In addition, MCP-1 has been shown not only to be expressed and secreted by breast cancer cells, but also to increase breast cancer cell invasiveness *in vitro* (37).

Studies by Wang and colleagues propose that the microenvironment of microscopic bone metastases in breast cancer is primarily composed of osteoblastic cells (7). The authors characterized the cellular composition of the bone niche in the presence of triple negative (Estrogen receptor (ER-), progesterone receptor (PR-) and HER/Neu -negative, MDA-MB-231) or estrogen-receptor positive (ER+, MCF-7) breast cancer metastases *in vivo*. They observed an increase of tartrate-resistant acid phosphatase (TRAP) -positive osteoclasts during the transition from indolent, non-proliferative micrometastases to the osteolytic cycle. In contrast, during the pre-osteolytic stage under 20% of niche cells surrounding the microscopic breast cancer bone metastases could be accounted to the osteoclastic lineage. Furthermore, the cathepsin K-positive osteoclasts were not in direct contact with the cancer cells. However, around 80% of the cells adjacent to the breast cancer micrometastases abundantly expressed ALP and around 50% of the cells were positive for Col1, both markers for cells of the osteoblastic lineage (7). Furthermore, compared to tumor-free bones there was an enrichment of ALP and ColI -positive cells in bones containing micrometastases, suggesting that osteoblasts facilitate breast cancer cell colonization in the bone environment. Importantly niche cells showed active features of osteogenesis including the expression of Runx2 and Osx, regulators of osteoblast differentiation, as well as active Wnt signaling. Breast cancer cell—osteoblast interaction was mediated via heterotypic adherens junctions using E-, and N-cadherins. Consequently, this interaction resulted in an enhanced mTOR activity in cancer cells and was associated with the transition from DTCs into overt metastases (7), suggesting a potential route of how osteoblasts could regulate breast cancer cell dormancy in the bone.

While Wang and colleagues propose that osteoblasts would rather initiate metastatic tumor growth in bone and/or facilitate escape from dormancy, recently published studies by Kolb and colleagues identified a subtype of osteoblasts—termed tumor educated osteoblasts (EOs)—that have a functional role in suppressing breast cancer growth (8). Upon contact with tumor cells a subpopulation of osteoblasts was educated by the cancer cells into an osteopontin^high^ and αSMA^low^ phenotype *in vivo*. To further characterize the EOs, MC3T3-E1 cells were incubated *in vitro* with conditioned medium from MDA-MB-231 or MCF-7 breast cancer cells. Upon cancer cell stimulation EOs demonstrated lower abundance of the inflammatory cytokine IL-6 and increased expression of ECM remodeling proteins such as matrix metalloprotease 3 (MMP3) and Col1. Furthermore, conditioned medium from EOs retarded the proliferation of both, the metastatic MDA-MB-231 and the estrogen receptor positive MCF-7 breast cancer cell line *in vitro* as a reduced number of breast cancer cells entered the S-phase of the cell cycle. These studies suggest that distinct subpopulations of osteoblasts could contribute differently to tumor cell dormancy (8).

## The Role of Osteoblasts During Bone Metastases Progression

Little focus has been put on investigating the interaction between osteoblasts and breast cancer cells during bone metastases progression, mainly due to the fact that osteoblast numbers decrease during the advancement of osteolytic disease. By analyzing the distribution of metastatic MDA-MB-435 breast cancer cells in female athymic mice over a time period of 1 h to 6 weeks, Phadke and colleagues observed that breast cancer micrometastases (<10 cells) resided in great proximity to osteoblastic cells, whereas the number of osteoblasts decreased as tumor burden increased (32). Also studies by Brown and colleagues report that the presence of tumor cells modifies the osteoblast-osteoclast ratio in the bone microenvironment, and that these changes largely depend on whether there is direct contact between bone and tumor cells (38). In these studies, the effect of tumor cells on osteoblasts was most profound prior to the initiation of osteolytic disease. Compared to naïve mice, osteoblast number per mm trabecular bone surfaces was significantly increased in tumor bearing mice prior to the onset of metastatic bone disease, followed by a decrease in the osteoblast/osteoclast ratio once osteolytic lesions were apparent. Interestingly though, a more detailed analysis of osteoblasts adjacent and distant to the tumor cells revealed that the number of osteoblasts distant from the tumor cells was increased compared to those in direct contact with the tumor (38).

These data suggest a key role of osteoblasts during the early stages of breast cancer bone metastasis. Several *in vitro* studies support this hypothesis. For instance, osteoblast conditioned medium can act as a chemoattractant for breast cancer cells. A 12% increase in cell migration was observed when MDA-MB-231 breast cancer cells were allowed to migrate toward medium conditioned by osteoblasts compared to control medium using the transwell migration assay (34). Using the wound healing assay, pre-osteoblasts (ALP^low^, OPN^low^, Runx2^high^, Osx^high^, CD166^high^) but not mature osteoblasts were shown to enhance the migration of MDA-MB-231 breast cancer cells (39). By using a vybrant cell adhesion kit the authors were also able to show that adhesion of MDA-MB-231 breast cancer cells to pre-osteoblastic cells was strongly increased when compared to undifferentiated cells or mature osteoblasts, suggesting that osteoblasts regulate early stages involved in metastatic breast cancer growth (39). Vice versa, data also suggest that breast cancer cells can stimulate the migration of mesenchymal cells, progenitors of osteoblasts (40). In contrast, a specific sub-type of osteoblasts [OPN^high^ and alpha-smooth muscle actin (αSMA^low^)] has been shown to retard breast cancer cell proliferation (8). In summary, these findings highlight that there is indeed an interaction between osteoblasts and breast cancer cells during the early stages of breast cancer bone metastases and that these communications could determine whether tumor cells undergo dormancy or whether they develop into overt metastases.

## Osteoblasts as Novel Target to Treat Bone Metastases—Bone Anabolic Treatment

**Figure 3:**
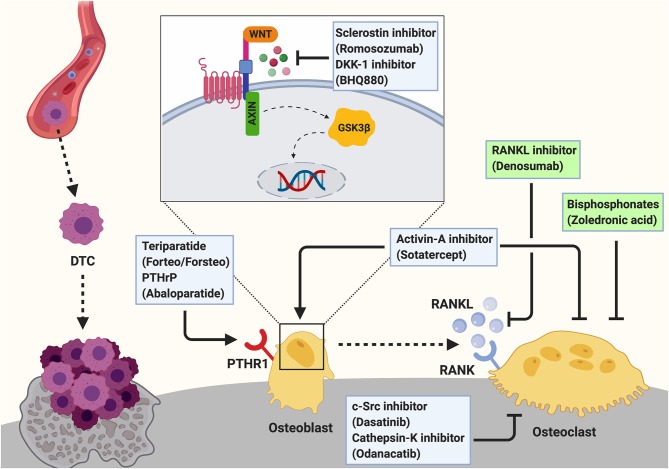
Targeting the osteogenic niche to treat breast cancer bone metastasis. Breast cancer bone metastases remain incurable once osteolytic lesions have developed. Palliative treatment often includes the administration of osteoclast-targeted, anti-resorptive agents including bisphosphonates (e.g., Zoledronic acid) or the anti-RANKL antibody Denosumab to prevent the cancer-induced bone resorption. These two agents are the only approved treatments for cancer induced bone disease (indicated by green box). Additionally, c-Src (Dasatinib) and Cathepsin-K (Odanacatib) inhibitors are under investigation for the treatment of breast cancer bone metastasis. As these anti-resorptive agents are not able to restore the cancer-induced bone loss, augmenting osteoblast function by anabolic treatments has been proposed as a potential therapeutic approach and several agents are investigated experimentally and/or in clinical trials. Bone anabolic treatments including the administration of a recombinant fragment of PTH (Teriparatide; Forteo/Forsteo) or Parathyroid hormone related protein (PTHrP; Abaloparatide) are approved for the treatment of osteoporosis. However, these drugs cannot be prescribed for patients with bone metastases. Another bone anabolic agent Romosozumab, an antibody against the Wnt signaling inhibitor Sclerostin, increases bone formation and bone mass by activating the Wnt pathway in osteoblasts. Similarly, Dkk-1 inhibitors (e.g., BHQ880) allow active Wnt signaling in osteoblasts thereby increasing osteoblast activity. Inhibition of Activin-A signaling has been shown to prevent cancer-induced bone destruction. Additionally, Activin-A inhibitors (e.g., Sotatercept) have been shown to stimulate osteoblastogenesis while decreasing osteoclast activity to promote bone formation. Hence, they could potentially be a novel approach for the treatment of cancer induced bone disease.

Advancements have been made in limiting progression of breast cancer bone metastasis and novel therapeutic agents are emerging (41). However, once osteolytic lesions have been developed, the disease remains incurable and treatment is restricted to palliative care. This often includes the administration of the anti-resorptive bisphosphonate Zoledronic acid, or of the RANKL inhibitor Denosumab to reduce the cancer-induced bone destruction (42–44) (Figure 3). Further experimental approaches to target osteoclasts in metastatic bone disease include Cathepsin-K and c-Src inhibitors (45, 46). However, these agents are not able to restore the cancer-induced bone destruction. Therefore, augmenting osteoblast function has been proposed as a potential approach to restore bone integrity in the context of metastasis-induced osteolytic lesions (47).

Osteoporosis is a debilitating disease that leads to loss of bone mass and ultimately results in fragility fractures (48), similarly as in cancer-induced bone disease. To date, three bone anabolic drugs are available in the clinic for the treatment of severe osteoporosis. Two of the drugs are based on the activation of the PTH receptor by an intermittent administration of a recombinant fragment of PTH (Teriparatide; Forteo/Forsteo) or Parathyroid hormone related protein (PTHrP; Abaloparatide) (49). Recently, the effect of PTH on breast cancer bone metastasis was investigated in two studies. A short term (5 days, daily) administration of PTH prior to intracardial injection of MDA-MB-231 breast cancer cells was shown to have no effect on tumor cell homing or growth in the hind limbs of mice (50). However, tumor burden was increased in other skeletal sites suggesting that PTH-mediated alteration of the endosteal niche renders different skeletal sites to cancer cell colonization (50). In contrast, an anabolic (4 weeks, daily) treatment of mice with PTH was demonstrated to prevent skeletal metastases and preserve bone architecture in orthotopic and intratibial breast cancer models (51). Despite different experimental design, which is likely to explain the different results, both studies demonstrate that alteration of the bone microenvironment and osteoblast function by PTH affect breast cancer bone colonization. However, the use of Teriparatide is not approved for use in patients with a history of primary or metastatic bone cancer (52).

The third bone anabolic agent is an antibody against the Wnt signaling inhibitor Sclerostin (Scl-Ab; Romosozumab) that increases bone formation and bone mass by activating the Wnt pathway in osteoblasts (49, 53). In clinical trials, sclerostin antibody treatment of women with postmenopausal osteoporosis increased bone formation, while bone resorption was decreased, leading to an increase in bone mineral density and a reduction of the fracture rate at several sites, including the hip and spine (54). Similarly, the bone anabolic and anti-resorptive effect of Scl-Ab was recently demonstrated in a pre-clinical mouse model of bone metastases (55). Importantly, Scl-Ab treatment not only reduced metastatic breast cancer burden *in vivo* but also protected from cancer-induced bone and muscle loss and increased survival of cancer-bearing animals (55). In addition, further agents targeting osteoblast differentiation and function have been investigated for the treatment of bone metastasis in various cancers, with potential benefits also for breast cancer-induced bone disease (41). These include for example inhibitors of Dickkopf 1 (Dkk-1) and Activin-A (Figure 3). Similar to sclerostin, Dkk1 antagonizes Wnt signaling in osteoblasts. Consequently, inhibition of Dkk1 resulted in increased bone formation and reduced osteolysis in a mouse model of multiple myeloma, highlighting the potential benefit as an osteoanabolic agent (41, 56). Inhibition of Activin-A signaling with a soluble activin receptor type IIA fusion protein (ActRIIA.muFc) has been shown to stimulate osteoblastogenesis, promote bone formation and to inhibit bone metastasis and prevent bone destruction in a murine model of breast cancer bone metastasis (57). Although more investigation is needed, these studies suggest that targeting the endosteal niche by bone anabolic treatments could be a future approach to treat osteolytic bone metastases.

## Cancer Associated Fibroblasts in Breast Cancer (Bone) Metastasis

As discussed in the previous sections, osteoblasts that originate from MSCs are increasingly recognized as therapeutic targets for breast cancer bone metastasis (8, 29). Another mesenchymal-derived, endosteal niche cell type with a potential to regulate the establishment and progression of bone metastasis includes fibroblasts. Although usually quiescent in normal tissue, fibroblasts acquire an activated phenotype during processes such as wound healing, tissue inflammation or fibrosis. Given the physiological role of fibroblasts, their involvement in tumor growth is apparent as cancers are considered as “wounds that do not heal” (58). Cancer-associated fibroblasts (CAFs), activated fibroblasts that are associated with cancer, are one of the most abundant stromal cell types in breast cancer and are associated with poor prognosis (59) (Figure 2).

The contribution of CAFs in cancer progression has been extensively reviewed elsewhere (60, 61). Briefly CAFs produce growth factors that contribute to disease establishment (e.g., hepatocyte growth factor (HGF), TGF-β, SDF-1, VEGF, IL-6) in addition to MMPs. All of these factors are well-known to affect several hallmarks of cancer (60, 61). Whereas, the contribution of CAFs to primary tumor growth is intensely investigated and defined, the origin and role of CAFs in the metastatic environment, especially in breast cancer bone metastasis, remain poorly defined (62). Within the next chapters we discuss evidence that supports a role of CAFs during the progression and establishment of breast cancer bone metastasis.

## Origin and Characterization of CAFs in the Tumor Microenvironment

The origin of CAFs in the tumor microenvironment remains to be elucidated, but they might be derived from resident fibroblasts (63), actively recruited bone marrow-derived cells (64) or cells that undergo epithelial-mesenchymal transition (EMT) (65).

Due to the phenotypical and functional heterogeneity of CAFs there are no unique markers to identify them but commonly used ones include αSMA, fibroblast-specific protein1 (FSP1 or S100A4), fibroblast activation protein (FAP), platelet derived growth factor receptors (PDGFRα/β), vimentin, and tenascin C (66–68). Several *in vitro* studies demonstrate that MSCs can differentiate into αSMA -expressing myofibroblasts upon cancer cell stimulation (66, 69). For instance, studies by Mishra and colleagues show that human bone marrow-derived MSCs can acquire a CAF-like, myofibroblastic phenotype upon prolonged stimulation with conditioned medium from MDA-MB-231 breast cancer cells. Importantly, these cells expressed CAF markers including αSMA, SDF-1, vimentin, and FSP as determined by immunofluorescence staining. Gene expression analysis revealed that cancer-conditioned medium upregulated the expression of CAF-associated genes including SDF-1, platelet derived growth factor α (PDGFα) and MMP9, suggesting that exposure to cancer cells induces hMSC differentiation into a CAF-resembling state (66).

## The Role of CAFs During the Establishment and Progression of Breast Cancer Bone Metastasis

CAFs are thought contribute to both, primary and secondary breast cancer through regulating processes such as breast cancer cell proliferation and stemness as well as ECM remodeling, production and stiffness (67). Furthermore, CAFs are involved in regulating cancer cell migration, invasion and distant metastasis (58, 60, 70). Certain survival pathways including the c-Src pathway are suggested to be detrimental for metastatic latency. Indeed, using a gene expression profiling Zhang and colleagues revealed a strong correlation between c-Src activity and bone metastasis [a Src response signature (SRS) (71). Further studies linked the SRS and the CAF-content of primary breast tumors to the likelihood of these tumors to relapse in bone (72). In these experiments the authors demonstrate that triple negative breast cancers with a high SRS (SRS+) and therefore a high preference to metastasize to bone, had increased expression of CXCL12/14 and IGF-1/2 when compared to SRS- tumors. Interestingly, CAFs were identified as the source of these cytokines rather than the tumor cells themselves. Consequently, the authors suggest that a high prevalence of mesenchymal cells including CAFs in the stroma of triple negative breast tumors would select for certain clones. These include in particular clones that grow well under the presence of CAF-derived cytokines including CXCL12 or IGF-1. This in turn would lead to a predisposition of disseminated tumor cells to colonize the bone marrow which has a higher abundance of stromal -derived CXCL12 and IGF-1 compared to other metastatic sites such as lung, liver and brain (72). Additionally, it has been demonstrated that fibroblasts isolated from different sites of breast cancer growth, including the breast, lung and bone, enhance the invasiveness of ER+ breast cancer cells in an IL-6 dependent way (73). Furthermore, CAF-induced ECM remodeling and altered tissue stiffness might contribute to tumor cell invasiveness, dissemination and/or metastasis. Studies by Madsen and colleagues demonstrated that hypoxia reduced periostin and αSMA expression in CAFs, two common markers that indicate CAF activation (67). HIFs mediate response to hypoxia and in these studies HIF-1α supported CAF-induced matrix remodeling and invasion. Prolyl hydroxylase domain-containing proteins (PHDs) are enzymes that target the alpha subunits of HIF complexes for degradation under normoxic conditions. Interestingly loss or inhibition of PHD2 suppressed CAF induced matrix remodeling and invasion *in vitro*. In a 4T1 mouse model, inhibition of PHDs reduced stiffness of primary 4T1 tumors as well as the development of spontaneous metastasis to lung and liver (67). The authors suggest that targeting PHD2 in CAF-enriched tumors, including breast cancer, may have beneficial effects on metastasis development. However, metastasis to bone was not assessed in this study. Besides being a highly vascularized tissue, the bone microenvironment is hypoxic and regional oxygen tensions vary depending on the level of cellularity, oxygen consumption and supply of oxygenated blood. Hypoxia and activation of HIF1α as well as HIF2α is known to contribute to tumor progression and metastasis in various organs including breast cancer (74), but besides the studies by Devignes and colleagues (31) little is known about the impact of hypoxia in breast cancer bone metastasis. Studies by Hiraga and colleagues demonstrated that increased HIF1 α expression in MDA-MB-231 breast cancer cells enhanced the colonization after intracardiac inoculation (75). These findings provide room to speculate a role of CAFs in promoting and/or regulating breast cancer bone metastasis (67).

## Conclusion/Perspective

Over the last decade significant progress has been made in understanding metastatic breast cancer growth in bone. However, the disease remains incurable once tumor cells are actively proliferating in bone. Many aspects, in particular the initial stages of bone metastasis, need to be investigated further in order to prevent disease establishment.

Research has recently focused on deciphering the early events of metastatic tumor growth in bone, including the entry and exit from dormancy or the transition from micro-metastases to overt metastases (7, 24, 27, 28, 76). Substantial evidence exists that cancer cells interact with cells of the bone microenvironment to render physiological processes and/or cell to cell communications in order to promote tumor cell maintenance, survival and proliferation in bone (3, 7, 34, 77–79).

One important component of the (bone) tumor microenvironment includes mesenchymal-derived cells including osteoblasts and fibroblasts or so called “endosteal niche cells.” Endosteal niche cells and in particular osteoblasts are increasingly appreciated as important components of the metastatic niche (8, 34, 35, 38, 39, 80, 81). However, their role in supporting tumor cell homing, dormancy and disease progression remains poorly defined. Unlike osteoclasts, the contribution of osteoblasts to breast cancer bone metastasis remained under-investigated over the last years. Nevertheless, recently published studies highlight their potential as novel cellular targets to prevent and/or treat breast cancer bone metastasis (7, 8, 29, 34, 38, 39, 75). In addition, the therapeutic importance of osteoblasts has been acknowledged with novel therapeutics including bone anabolic agents such as PTH and anti-sclerostin antibody (51, 55). However, a detailed characterization of how bone anabolic agents modify the composition and/or location of the endosteal niche as well as potential consequences on tumor cell colonization and metastatic outgrowth remains to be performed. In addition, further research is needed to investigate whether stimulating osteoblast activity would result in the repair of osteolytic bone lesions. This also raises the question whether a combination of anti-osteolytic and bone anabolic therapy would be beneficial for the treatment of breast cancer bone metastasis.

Another mesenchymal-derived cell type in the bone microenvironment includes fibroblasts, which transform into CAFs upon the presence of disseminated tumor cells. CAFs are known to create a tumor permissive environment by influencing nearly all hallmarks of breast cancer (60, 61, 82). The role of CAFs in promoting tumor growth is evident, they release growth factors, stimulate angiogenesis, proliferation, migration as well as ECM remodeling. However, these findings are primarily derived from research that has been limited to the primary tumor. In contrast, little is known about their role in the metastatic environment. Especially the contribution of CAFs to the establishment and progression of breast cancer bone metastasis is poorly defined (64–67). Another open question remains the origin of CAFs in the metastatic (bone) environment. Identifying the origin of CAFs would provide targets to suppress their tumor growth-supporting function.

In summary, a deeper understanding of the interaction between the endosteal cell compartment and disseminated breast cancer cells will be needed to develop more successful treatment for breast cancer bone metastases. Research techniques to investigate cell-cell interactions, especially *in vitro*, have significantly improved over the last years. Nevertheless, our ability to track and visualize these interactions *in vivo* remains limited. This highlights the need to improve our model systems as well as imaging techniques to increase our knowledge about the interaction between tumor cells and cells of the microenvironment. Consequently, this will aid to elucidate the mechanisms of how osteogenic cells suppress or promote metastatic growth in bone and would provide novel therapeutic targets that could be used to maintain disseminated tumor cells in a dormant state or to completely prevent dissemination/colonization in the bone.

## Author Contributions

MTH and HT reviewed the literature and wrote the manuscript. DS reviewed the literature and prepared the figures.

### Conflict of Interest

The authors declare that the research was conducted in the absence of any commercial or financial relationships that could be construed as a potential conflict of interest.
